# Composing only by thought: Novel application of the P300 brain-computer interface

**DOI:** 10.1371/journal.pone.0181584

**Published:** 2017-09-06

**Authors:** Andreas Pinegger, Hannah Hiebel, Selina C. Wriessnegger, Gernot R. Müller-Putz

**Affiliations:** 1 Institute of Neural Engineering, Graz University of Technology, Graz, Austria; 2 Institute of Psychology, University of Graz, Graz, Austria; Universita degli Studi di Palermo, ITALY

## Abstract

The P300 event-related potential is a well-known pattern in the electroencephalogram (EEG). This kind of brain signal is used for many different brain-computer interface (BCI) applications, e.g., spellers, environmental controllers, web browsers, or for painting. In recent times, BCI systems are mature enough to leave the laboratories to be used by the end-users, namely severely disabled people. Therefore, new challenges arise and the systems should be implemented and evaluated according to user-centered design (USD) guidelines. We developed and implemented a new system that utilizes the P300 pattern to compose music. Our Brain Composing system consists of three parts: the EEG acquisition device, the P300-based BCI, and the music composing software. Seventeen musical participants and one professional composer performed a copy-spelling, a copy-composing, and a free-composing task with the system. According to the USD guidelines, we investigated the efficiency, the effectiveness and subjective criteria in terms of satisfaction, enjoyment, frustration, and attractiveness. The musical participants group achieved high average accuracies: 88.24% (copy-spelling), 88.58% (copy-composing), and 76.51% (free-composing). The professional composer achieved also high accuracies: 100% (copy-spelling), 93.62% (copy-composing), and 98.20% (free-composing). General results regarding the subjective criteria evaluation were that the participants enjoyed the usage of the Brain Composing system and were highly satisfied with the system. Showing very positive results with healthy people in this study, this was the first step towards a music composing system for severely disabled people.

## Introduction

Brain-computer interfaces (BCIs) are useful tools to provide communication without the need of any voluntary muscular control. A BCI can be an assistive device for people who are suffering from severe disabilities, i.e., who cannot communicate via the normally available channels due to motor degeneration or brain damage [1]. The so-called P300 event-related potential (ERP) is a prominent brain signal for BCI-control and is often assessed non-invasively by measuring the electroencephalogram (EEG). Farwell and Donchin [2] developed the first P300-based BCI application utilizing the so-called oddball paradigm where approx. 300ms after the presentation of a rare stimulus between frequently presented standard stimuli a positive deflection in the EEG occurs [3]. The P300 was elicited by randomly flashing the rows and columns of a 6 × 6 matrix containing the letters of the alphabet and numbers between 0–9. Volunteers were asked to count the flashings of the symbol to be selected and to ignore the highlighting of the other characters. Almost all existing BCIs attempting to evoke the P300 pattern visually are using this method. This type of BCI allows writing characters and letters or selecting commands on a computer screen. Based on the oddball principle, also auditory [4] and tactile [5] P300-based BCIs were developed and evaluated with healthy as well as severely disabled people, e.g., [6–9]. It has been shown that with a P300 BCI it is possible to spell, browse the internet, control a smart home, and drive a wheelchair [10–12]. Also applications for entertainment have been developed [13, 14].

One example for an application which allows the users to paint pictures and thereby express their creativity is the so-called Brain Painting application. This application was designed by the German artist Adi Hösele in cooperation with the Institute of Medical Psychology and Behavioural Neurobiology at the University of Tübingen [15]. A P300-based BCI is the basis of the Brain Painting system. With a special P300 matrix, it is possible to select the color, grid size, object size, transparency, and other features which allow painting pictures on a virtual canvas. Various studies have been conducted with the Brain Painting application demonstrating that it is possible for healthy people as well as for severely disabled people to paint pictures [16–18]. Furthermore, the Brain Painting system was used by several severely disabled painters in their homes over a long time period and these painters had several exhibitions in different countries [17]. The development of the Brain Painting application was based on a user-centered design (USD) approach according to the ISO 9241–210 norm. UCD is becoming more and more important in BCI research. Many studies have already been published regarding this topic [19–22]. According to Kübler et al. [23] a BCI system for communication and control developed by UCD standards is evaluated and improved by three main factors, namely effectiveness, efficiency and satisfaction.

Besides painting pictures, another possibility for creative expression is to make music. Utilizing the EEG to make music was first introduced by Adrian and Matthews in 1934 [24]. They implemented a sonification of the EEG signals. The first attempt to really compose a musical piece using EEG was performed by Lucier et al. [25] in 1965. Other composers, like Rosenboom [26] and Teitelbaum [27], followed. All these early so-called brain-to-music interfaces are based on sonification of the EEG signals. The first attempt to assess the performer’s attention with the EEG and make parameter-driven music by detecting selective attention was introduced by Rosenboom in 1990 [28]. Fifteen years later Miranda and Boskamp introduced the brain-controlled piano [29]. They gave generative rules to the most prominent frequency bands in the spectrum of the EEG. Additionally, the system measured the complexity of the EEG signals to modulate the tempo and dynamics of the music. Wu et al. proposed a direct parameter mapping method to translate characters of the EEG into musical notes which is based on the power law of brain activities and music [30]. Later this method was extended for deriving a quartet from multichannel EEG [31]. Daly et al. developed and evaluated an affective brain-computer music interface for modulating the affective states of its users [32]. Their system attempts to modulate the users current affective state by playing music which is generated by an algorithmic music composition system and a case-based reasoning system. An overview about brain-to-music interfaces is given in the book: “Guide to Brain-Computer Music Interfacing” [25].

Utilizing the P300 component of the EEG to compose music was introduced by Grierson et al. [33]. They arranged different tone pitches, between A1 and G5, on a P300 spelling matrix. In a pilot study, five users were asked to select the C major notes, i.e., *c*’’’, *d*, *e*, *f*, *g*, *a*, *b*, *c*’’’’. Four of the tested five subjects could finish the task with an accuracy rate of 75% or above.

Our Brain Composing system is based on the hypothesis that it is possible to effectively compose music via BCI without constraints. Therefore, we combined two powerful systems, a P300-based BCI with a music composing software. The BCI allows the user to control the composing software completely by concentrating on the elements of the P300 matrix. In addition to the suggested USD approach, in our opinion, a BCI system for disabled people has to be developed in two steps: first, the system has to be tested and evaluated with healthy subjects and improved according to the suggestions of that user group. In a second step, the system has to be evaluated with the disabled users and adapted according to their feedback. This two-step method allows solving error and usability problems of the system before the intended end-users work with it for the first time. The objective of this strategy is to avoid that severely disabled people become demotivated by initial problems.

A pilot study, addressing the usability of the Brain Composing system, showed positive results [34]. Five healthy participants took part in the pilot study. Their task was to copy-compose a given melody with the Brain Composing system. A minimum of 42 selections were necessary to finish the task. Three participants completed the task with accuracies between 77.8 and 95.7% and two participants were able to copy-compose more than half of the melody correctly.

The aim of the current study is to test our hypothesis and therefore, to investigate accuracy and user-acceptance of the Brain Composing system. User acceptance was determined with visual analogue scales, user experience questionnaires, and workload assessments. We evaluated the Brain Composing system with 17 healthy volunteers with musical background and one professional composer with at least 40 years experience in composing. They were asked to perform several tasks with the system and answer several questionnaires before and after the usage of the Brain Composing system. Tasks were a copy-spelling task, two copy-composing tasks and a free-composing task. This study was the proof of concept before testing the system with disabled people.

## Materials and methods

The designed Brain Composing system consists of three parts: the EEG acquisition system, the P300 control software, and the music composing software. For signal acquisition, we used a gel-less biosignal acquisition system. Additionally, a universal P300-based BCI control system [11] was connected to a powerful, open-source music composing software (MuseScore 1.3, https://musescore.org).

### Data acquisition

EEG signals were recorded with the Mobita (Twente Medical Systems International B.V., Oldenzaal, the Netherlands) biosignal amplifier, which transmits signals with 24 bit resolution via Wi-Fi wireless technology. The electrodes consist of small cotton pieces, connected to silver chloride pellets. The cotton is soaked in tap water prior to the measurement. The ground electrode is connected to a tap water soaked, conductive wrist band. The amplifier internally creates an average reference out of all used electrodes. Therefore, a real reference electrode is not required. This system ensures high usability [35]. EEG was recorded from six scalp electrodes (Fz, Cz, Pz, PO7, PO8, Oz) placed according to the extended international 10–20 system, with a sampling rate of 250 Hz.

### P300-based BCI control system

The used P300-based BCI control system is a further development of a system which was introduced in [36] and has been used for various studies, e.g., [11, 35, 37]. One of the main features is that it is a distributed system, i.e., a C-code written part is used for the stimulation, Matlab (The MathWorks, Natick, USA) is responsible for the signal processing, and another C-coded program handles the signal acquisition [38]. All the different parts are connected via a TCP network. The used data acquisition system delivers raw signals. Therefore, we used a 4*^th^* order Butterworth band-pass filter with cut-off frequencies of 1 and 15 Hz. As described in [11], different stimulation matrices are possible. In addition to the described method in [11], new ways to change the content of the P300 matrix and to control an external application were implemented. The content of the P300 matrix is stored in a JSON (javascript object notation) file. JSON is a lightweight data-interchange format. A JSON file can include the information for multiple matrices. The transition between different matrices is implemented by means of cross-links, i.e., every matrix has a unique name and can be called by an element of another matrix. In sum, every JSON matrix item consists of four parts: a symbol that is shown in the matrix, a value that is sent to the external application by key-press simulations, a cross-link element that can contain the name of another matrix, and finally a selectable element that indicates whether the symbol should change the color when it was selected. This implementation enables the user to control entire programs with the P300-based BCI.

Additionally, we implemented a dynamic stopping strategy that classifies the data after every flashing sequence, i.e., all rows and columns flashed once. If the classification result had been identical three times in a row, the corresponding element was marked yellow in the matrix, printed out in the bottom line, and sent to the controlled application. Therefore, the minimal number of highlighting sequences was three, cf. [12]. If the defined maximum number of flashing sequences was reached without having a final result, the stimulation was reset and started again.

### Music composing software

For the Brain Composing system, we connected the P300-based BCI control system with the music composing software MuseScore (https://musescore.org) version 1.3. This open-source music composing software provides an easily and commonly used environment to create high-quality western musical scores. Music can be composed for many different instruments, e.g., string instruments, piano, or brass instruments, by combining note lengths and note pitches. Additional features like rests, slurs, accords, and many more are also available. Sheets of music can be saved and exported in different media file formats, like MP3 or MIDI. However, the main reason why we decided to use this composing software is that an integrated shortcut manager allows creating shortcuts with different key combinations for nearly every possible command. In this way, all important control functions of the MuseScore software can be directly called via keyboard shortcuts.

### The composer control method

By selecting the MuseScore item in the menu bar of the P300-based BCI control application, the MuseScore application is started. At the same time, the P300-based BCI control application displays the main Brain Composing matrix consisting of four cross-link elements: “New”, “Open”, “Save”, and “Compose”. By selecting one of these first three elements, the MuseScore program opens the new, open, or save dialog window and the matrix changes to a matrix filled with Latin letters and control elements to create, open, or save a sheet of music. By selecting the “Compose” element, the user can directly start to compose music. Composing elements are displayed in the matrix and the last used sheet of music is shown in the MuseScore window.

**Fig 1:**
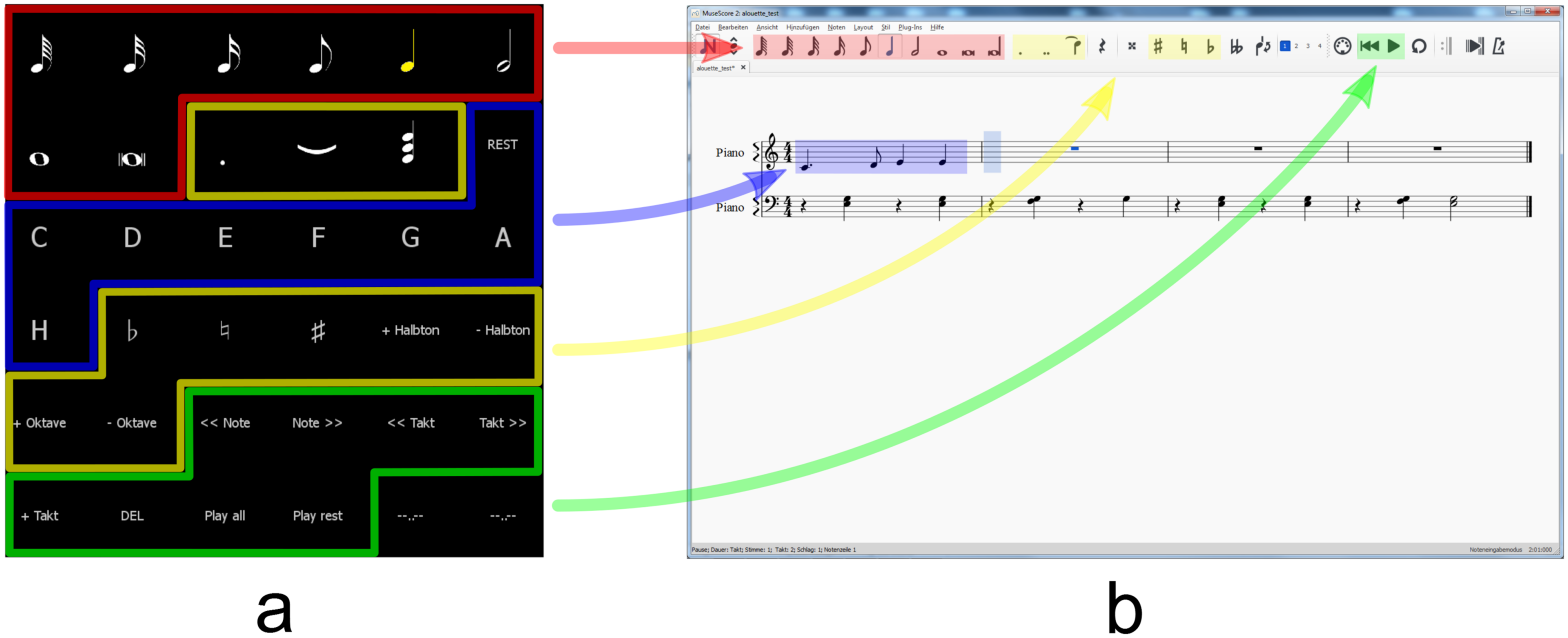
Brain Composing P300 matrix. Sketch of the P300 matrix and the corresponding commands in MuseScore. (a) Screenshot of the black and white P300 stimulation matrix; (b) Screenshot of the MuseScore window. All colored areas are inserted to visualize the different commands for the reader and were not shown during the study.

To insert a note into a given sheet of music, first the note length has to be selected, see Fig 1 red arrow. The currently selected value is indicated by yellow color in the P300 matrix, see Fig 1(a). Extra features for the note, like accidentals, dot, slur or chord, can be selected, see Fig 1 yellow arrow. Accidentals and dots are just applied to the note pitch that is selected subsequently, whereas the slur and chord function remain activated, marked with yellow color until selected again. Finally, to add a note, a pitch has to be selected, see Fig 1 blue arrow. Afterwards, the selected note is played and the user sees the note on the sheet of music. Errors can be corrected by deleting the note. Two elements (”play all”, “play rest”) are available to play the composed melody, see Fig 1 green arrow. Various other elements are available, e.g., to navigate back and forth between notes or bars and to change the pitch in steps of one octave.

### Study design and procedure

**Fig 2:**
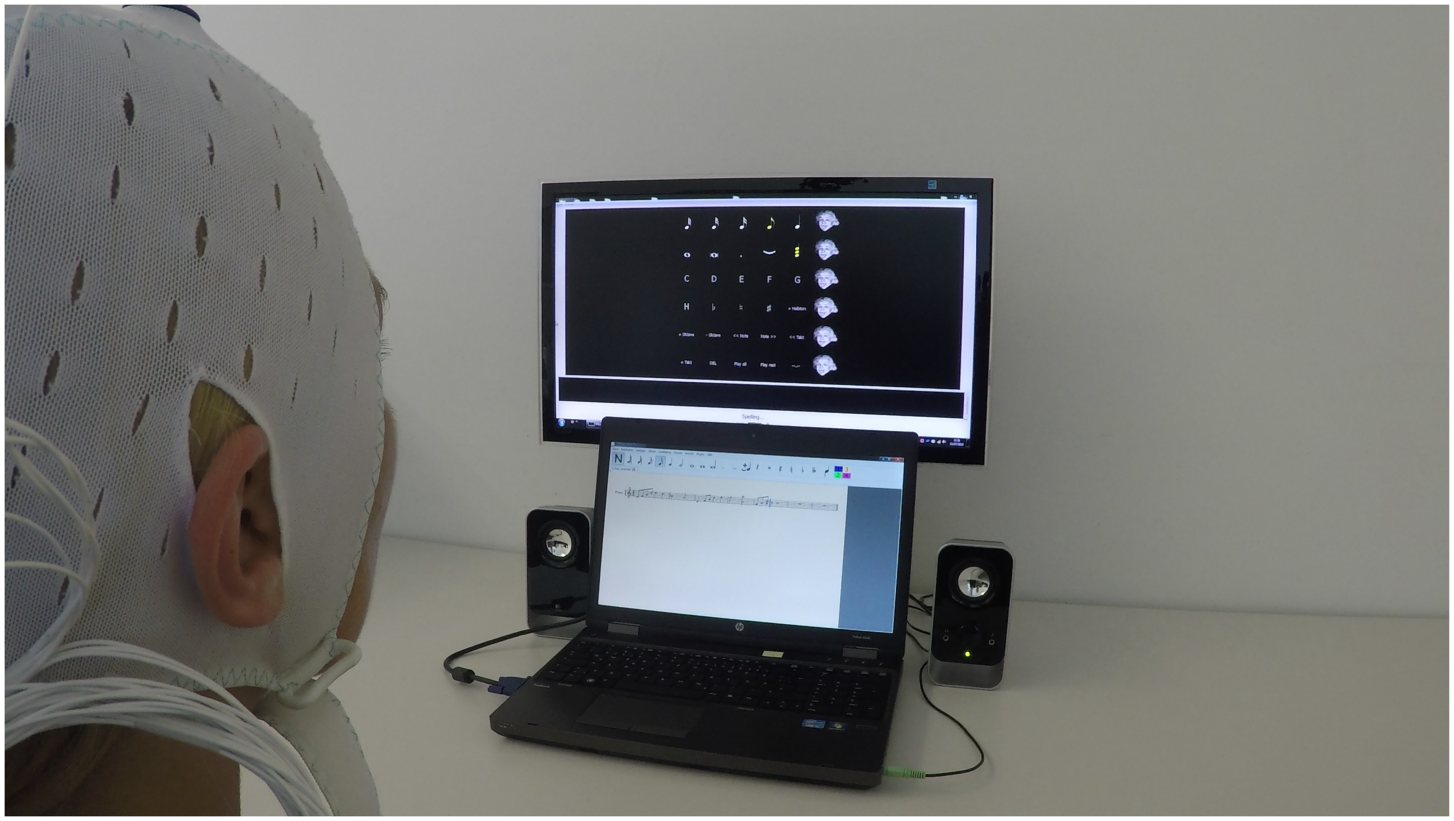
Brain Composing setup. The upper screen shows the P300 stimulation matrix and the bottom screen shows the music composing software.

We evaluated the new Brain Composing system with eighteen participants in terms of efficiency, effeciveness and satisfaction. During the performed experiment, participants were seated in a comfortable chair approximately 70 cm away from two computer screens centered in front of them, see Fig 2. The upper screen displayed the P300 matrix used to control the music composing software, which was shown on the bottom screen when activated. The bottom screen remained black during the calibration and copy-spelling tasks.

#### Participants

Seventeen healthy, non-professional musicians, hereinafter called non-professional participants, (5 female, mean age: 27.12, SD:8.54 years) took part in the study (16 right-handers, 1 left-hander). Twelve participants were naive to BCI, four had experience with BCI (not P300-based), and one had taken part in the Brain Composing pilot study. All participants disavowed any history of neurological or psychiatric disease and hearing impairment, and had normal or corrected-to-normal vision. They gave written, informed consent before the experiment. The study was approved by the Ethic Committee of the Medical University of Graz, Austria.

Before the main experiment participants had to fill out a questionnaire covering different aspects of musical training, instruments and demographic information. All participants had played at least one instrument and/or sang (mean duration: 15.18, SD:5.83 years), and had received instrumental or vocal training in the past (mean duration: 10.74, SD:5.83 years). Four participants were still taking instrumental lessons. Twelve participants were playing their instrument/singing regularly (mean 5.25, SD:3.79 hours/week), five did currently not play/sing. Six participants had been playing/singing exclusively solo, eleven had additional experience in playing/singing in a band, orchestra or choir. All participants were able to read music notes. Apart from instrumental or vocal lessons, they had received musical training to a varying degree. However, none of them worked as a professional musician or composer. Nine participants stated that they did not compose music, eight composed music. Six participants reported to use composing software, three of them had used MuseScore before. The participants considered themselves as moderately to highly musical (M:7.55, SD:1.65), indicated by a score between 0 and 10 (0 = “not musical at all”, 10 = “highly musical”).

One professional musician and composer, hereinafter called professional composer, (68 years old, right-hander, BCI naive) took part in the study. He has played clarinet for 58 years and had received instrumental training for 20 years. He had studied clarinet, composition and orchestral training at the University and had been teaching music as a professor for many years. He has been working as a free-lance composer for more than 10 years and has created numerous compositions. He composed up to 10 hours/day and played clarinet 2 hours/day. He worked with professional computer software but had not used MuseScore before.

We performed the evaluation of the Brain Composing system separately for the non-professional participants and the professional composer to investigate related differences.

#### Calibration

For calibration, a 6 × 6 matrix, consisting of the letters of the German alphabet, the numerals 1–7, and three other commands, was used. Calibration was performed with 15 highlighting flashes per row and column, with a flash duration of 50 ms and an inter-stimulus interval (ISI) of 125 ms. Elements of the matrix were highlighted with famous faces [39]. Each block of sequences was followed by a four seconds pause. Participants were asked to copy-spell six symbols (”H3P5FU”), which were equally distributed over the matrix. At the beginning of each block, the target element was marked yellow in the matrix for two seconds. Participants were asked to focus their attention on the target and to mentally count the number of times the symbol was highlighted. Accuracy was calculated for every flashing sequence with a leave-one-letter-out cross validation. The calibration was successful when the accuracy was higher than 70% at any number of sequences.

#### Main experiment

**Fig 3:**
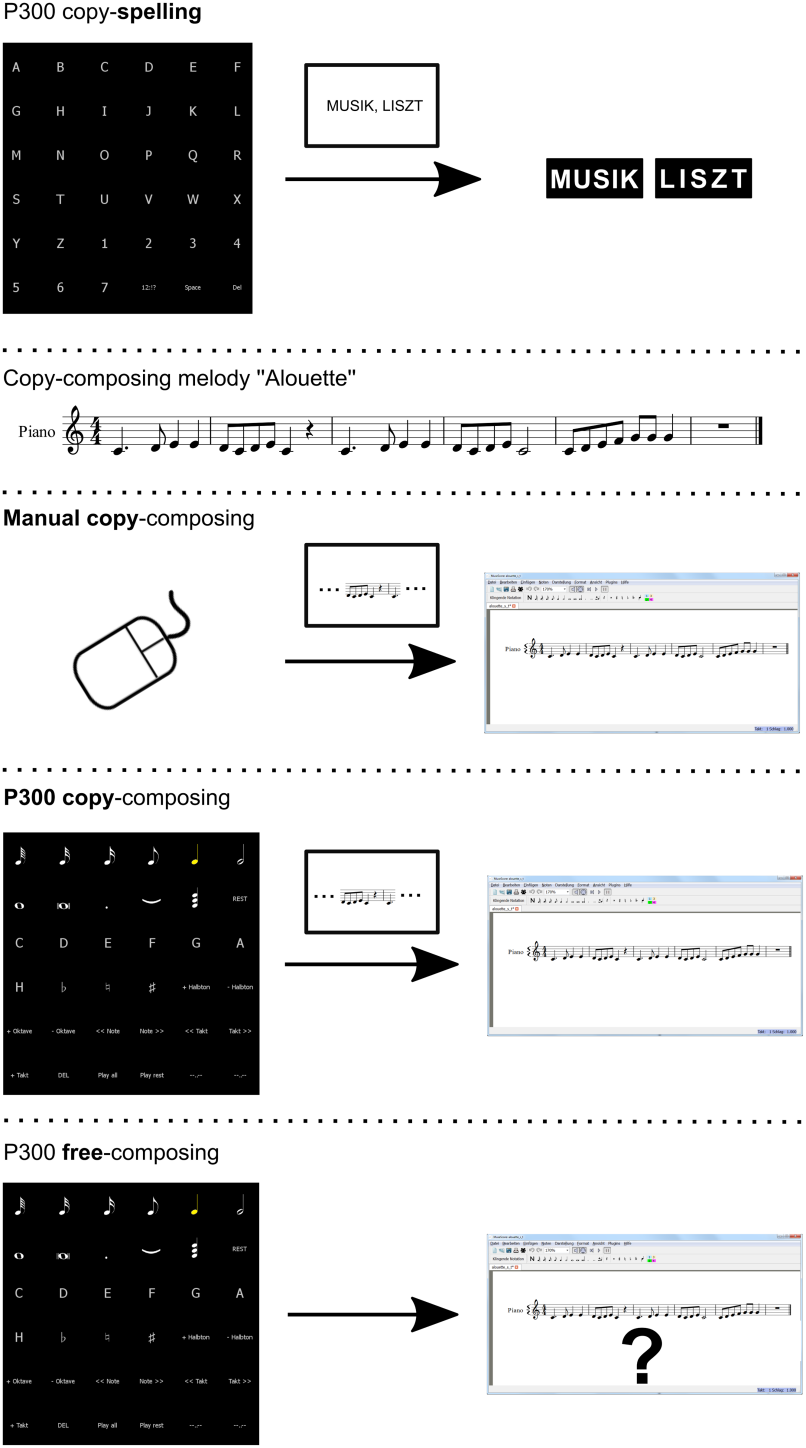
Sketch of the tasks. First row: Task 1 was to copy-spell “musik” and “liszt” with the P300-based BCI. Second row: The participants had to copy-compose the first six bars of the well-known French Canadian children’s song “Alouette”. Third row: Task 2 was to manually, i.e., by mouse-clicks, copy-compose the melody of Alouette. Fourth row: Task 3 was to copy-compose the melody of Alouette with the P300-based BCI. Fifth row: Task 4 was to compose free for 30 minutes.

After the calibration, participants had to fulfill four different tasks: a copy-spelling, a manual copy-composing, a P300-based BCI copy-composing, and a free-composing task, see Fig 3.

The copy-spelling task consisted of copy-spelling the words “MUSIK” (Eng. “MUSIC”) and “LISZT” (the name of a famous Austrian composer), see Fig 3, first row. The word to spell was inserted in the bottom line of the computer screen, below the P300 matrix. Stimulation parameters were equal to the calibration except the number of flashing repetitions, which were dynamically stopped. In case of an error, participants were instructed not to correct it but to proceed with the next selection. In the copy-composing task, participants were asked to copy-compose the first six bars of the well-known French Canadian children’s song “Alouette”, see Fig 3, second row. The melody was printed on a sheet of paper and placed at the top of the bottom monitor, thus located in the middle of the two screens. First, participants were given a verbal instruction how to control the composing software, insert music notes via the P300 matrix by mouse clicks, and get familiar with the application. Afterwards, they were asked to copy-compose the given melody via the P300 matrix by mouse clicks, see Fig 3, third row. In case of mistakes, further explanations were given how to control the music composing system.

For the P300-based BCI controlled copy-composing task, see Fig 3, fourth row, the pause after each block of sequences was set to 10 seconds in order to give the participants sufficient time to prepare for the next selection. Additionally, the participants were instructed to briefly state each element they intended to select before the next block of flashes started. Errors and false intentions were corrected via spoken commands of the experimenter. Intended and actual selections were noted in a protocol. The task included first selecting the “Compose” element in a 3 × 6 matrix with the elements “New”, “Open”, “Save”, and “Compose”. All other fourteen elements were filled with a meaningless symbol (”–,,–”). When the “Compose” element was selected, the matrix switched automatically to the 6 × 6 “composing” matrix and the music composing software was opened on the bottom screen with a prepared empty music sheet. After inserting all notes correctly, participants were asked to select the element “play all”. In total, 41 selections were required to complete the task. The task was aborted when the participants reached a number between 62 and 70 selections. This number varies because the task was aborted in this range when the user had no chance to finish.

After copy-spelling and copy-composing, participants could compose an individual melody (free-composing task), see Fig 3, fifth row. They were given a maximum of 30 minutes but they were also able to stop earlier. The stimulation parameters were identical to the copy-composing task. The participants again had 10 seconds time between each block of sequences to think about the next step, i.e., the next note length, pitch, feature. During this part of the experiment, they were no longer instructed to verbally state the symbols they intended to select but to say “false” in case of a misclassification, i.e., if the symbol they had focused on was not selected. Misclassifications were again noted in a protocol to calculate accuracies for the different tasks afterwards. Accuracies are defined as the ratio of the sum of correct selections to the sum of made selections for the copy-spelling, the copy-composing, and the free-composing tasks.

### Acquisition of behavioral data

In the present study, participants had to fill out several questionnaires covering their motivation, mood, fatigue, workload and user experience. In the following section, the used questionnaires are introduced in detail.

#### Motivation, mood, fatigue

Visual analogue scales have been used in many BCI studies, e.g., [20, 22, 40], and have been shown to be reliable and valid in measuring emotions or attitudes. The participants were asked to indicate their motivation, mood and fatigue on a VAS. Each VAS consists of a 10 cm long horizontal line with the anchor points 0 and 10 (0 = “not at all motivated”/ “bad mood”/ “not at all tired”, 10 = “highly motivated”/ “very good mood”/ “very tired”). Participants were asked to mark the position on the line which best represented their motivation, mood, or fatigue. Motivation was assessed before the experiment, mood and fatigue before and after the experiment. Pre- and post-values of mood and fatigue were compared with a paired sample t-test, respectively.

#### Workload

To assess subjective workload an electronic version of the NASA Task Load Index (NASA-TLX) [41] was administered. The NASA-TLX is a well validated instrument for workload assessment [42] also used in BCI research [21, 23, 43]. The NASA-TLX is a multi-dimensional scale used to estimate subjective workload on six dimensions: mental demand, physical demand, temporal demand, performance, effort, and frustration. Each of these factors is rated on a 20-step bipolar rating scale with a score ranging from 0 to 100 and anchor descriptors such as “high/low”. In a second step, participants indicate in 15 pairwise comparisons which factor contributed more to their subjective workload. The number of times a factor is chosen as more relevant is the weighting of the factor for the given task. By this weighting procedure, a global workload score is yielded (ranging from 0 to 100, a high score indicating a high workload), and the relative contribution of each factor to the total workload is identified (the highest possible score for each factor is 33.3).

#### User experience

To evaluate user experience (UX), the user experience questionnaire (UEQ) was administered [44]. The UEQ was developed to assess UX in an easy and immediate way, covering both pragmatic and hedonic aspects. It has been used to assess UX for a variety of software products, e.g., [45, 46] and was used in a recent BCI study [21]. The UEQ consists of 26 bipolar items rated on a 7-point semantic differential scale. The single items are transformed to the range from −3 to +3 and are assigned to six subscales: attractiveness, perspicuity, efficiency, dependability, stimulation, and novelty. Values above 0.8 indicate a positive impression, values below −0.8 a negative impression and values between −0.8 and 0.8 a neutral impression. The score of each subscale is calculated by averaging the rating of the corresponding items. The obtained subscales can further be grouped into three categories: attractiveness, use quality, and design quality. Attractiveness is a pure valence dimension, describing a person’s general attitude towards a product. Use quality reflects pragmatic quality aspects (average over the subscales efficiency, perspicuity and dependability) and design quality describes hedonic quality aspects (average over the scales novelty and stimulation).

In addition, participants completed a custom-made usability questionnaire (UQ) gathering further information about user satisfaction with the Brain Composing system, and rated their overall satisfaction, enjoyment and level of control on VAS (ranging from 0 and 10) after the experiment (0 = “not at all satisfied”/ “no enjoyment at all”/ “no control”, 10 = “absolutely satisfied”/ “absolute enjoyment”/ “absolute control”).

## Results

A video that demonstrates how the Brain Composing system works is available: S1 Video.

### BCI effectiveness and efficiency

**Fig 4:**
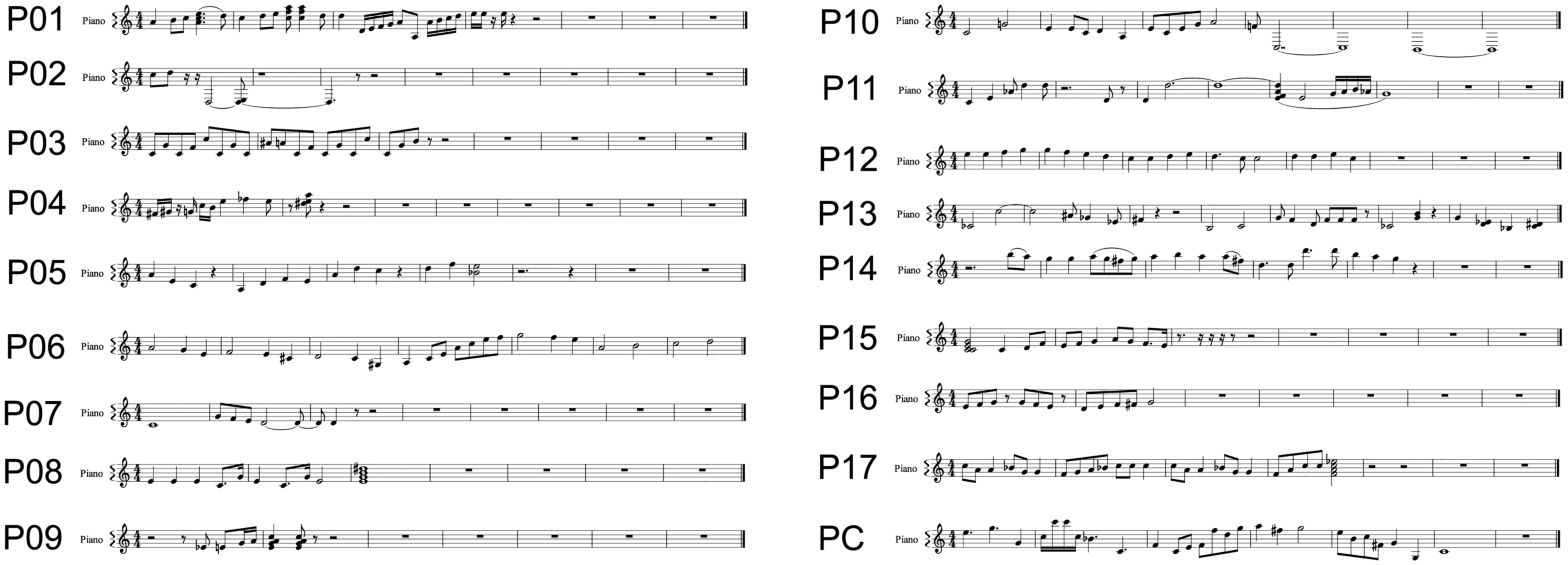
Accuracies of the different tasks. The accuracies of the copy-spelling, copy-composing, and the free-composing tasks are shown. P1-17 represent the non-professional participants and PC is the professional composer. Asterisks indicate that the participant did not finish the copy-composing task. The red dotted line indicates the 70% accuracy limit. Below that limit a BCI could not be used satisfactorily.

A comparison of the different accuracies per participant and task is shown in Fig 4. The accuracy has to be higher than 70% to be sufficient, cf. [47–50]. This threshold value is marked by a red dotted line in Fig 4. The non-professional participants’ (N = 17) average copy-spelling accuracy was 88.2 (SD:16.3)% in a range between 60 and 100%. The average time to spell one word (5 letters) was 77 (SD:6.8) seconds with a break of 6 seconds between the letters. For two participants, the task was unclear at the beginning. Their accuracy increased from 20% for the first word to 100% for the second word. Calculating the accuracy without these two participants (N = 15), the average accuracy would be 92.0 (SD:13.2)% instead.

The professional composer needed 73 seconds and 66 seconds to spell the two words with an accuracy of 100%.

Thirteen non-professional participants finished the copy-composing task with an average accuracy of 88.6 (SD:8.2)%. On average, they needed 54 (SD:9) selections to finish the task. With a pause of 11.5 seconds between the selections, the average time was 21:23 (SD:3:38) minutes. Four participants did not finish the task, because the task was aborted between 62 and 70 selections when the participants had no chance to finish it within 70 selections. However, at the end of the task only two participants were more than 10 steps away from finishing the composition. One participant copy-composed the given melody without any error. Six out of 17 participants composed the given melody with fewer than four errors. The professional composer composed the given melody with an accuracy of 93.6% in 20 minutes. He needed 47 selections.

**Fig 5:**
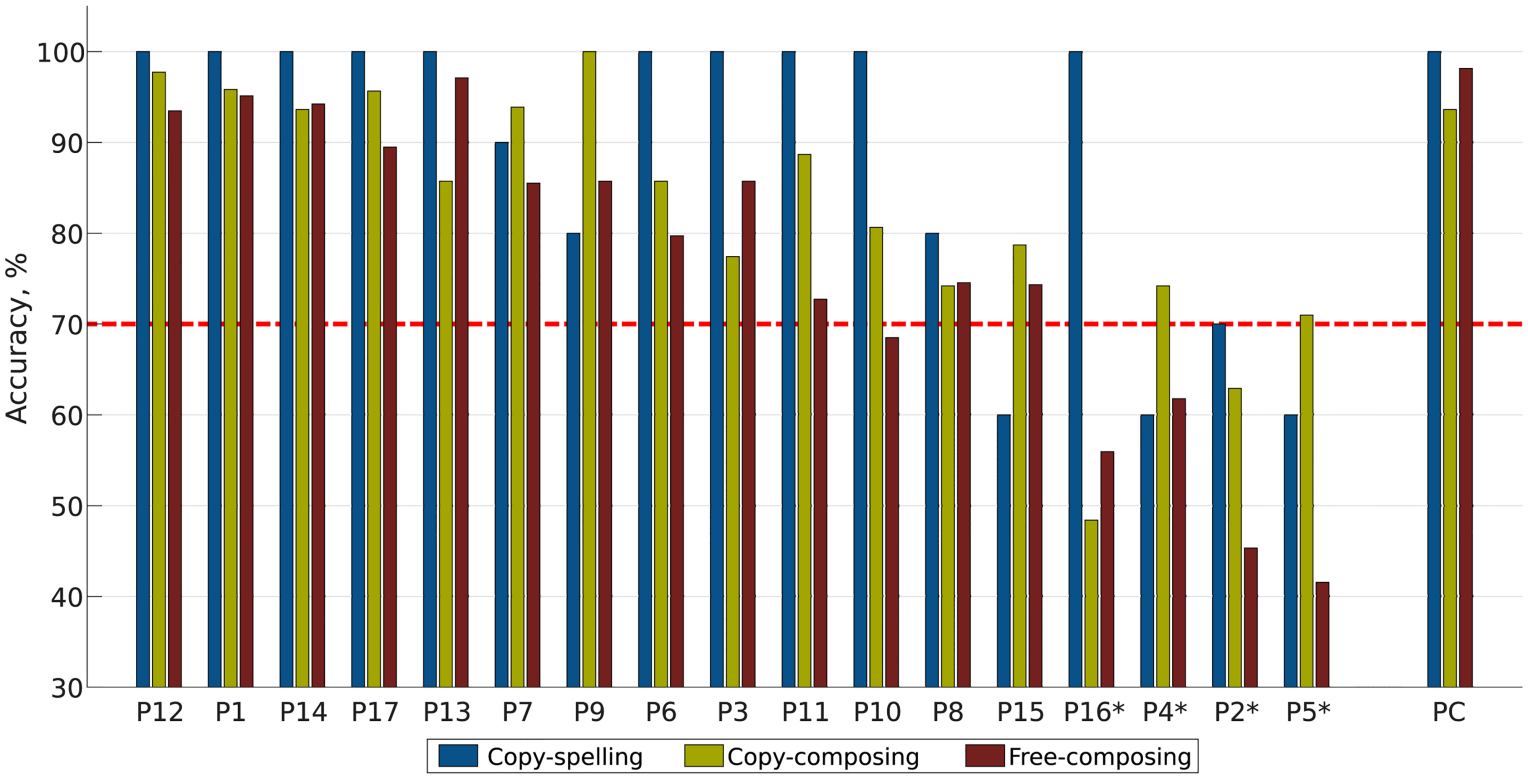
Participants’ free compositions. The non-professional participants’ (P1-17) and the professional composer’s (PC) musical pieces.

Thirteen non-professional participants used the full length of 30 minutes to compose their own melody. The four participants who did not used the whole 30 minutes, stated that they composed what they wanted to achieve. All the composed pieces of music are shown in Fig 5. To hear the compositions please use S1 Music. The average classification accuracy of the non-professional participants was 76.5 (SD:17.2)%. If the participants that could not finish the copy-composing task were excluded, the average accuracy would increase to 84.3 (SD:9.6)%.

The non-professional participants composed, on average, 17.9 (SD:6.9, range: 6–31) notes during the free-composing run. For this, they needed, on average, 4.3 (SD:2.3, range 2.4–10.7) selections per note. Consequently, the participants made 2.4 selections per minute (SD:0.21) with an inter-selection pause of 11.5 seconds. On average, they needed 1 hour and 32 minutes to fulfil all tasks plus the calibration with a standard deviation of 13 minutes. This period also includes pauses between the tasks. During that time the participants made, on average, 132 (SD:18) selections with the BCI.

The professional composer composed only fourteen minutes freely. However, he had an accuracy of 98.1%, composed 26 notes, needed 2.1 selections per note, and made 3.9 selections per minute.

### Behavioral data

#### Visual analogue scales

**Fig 6:**
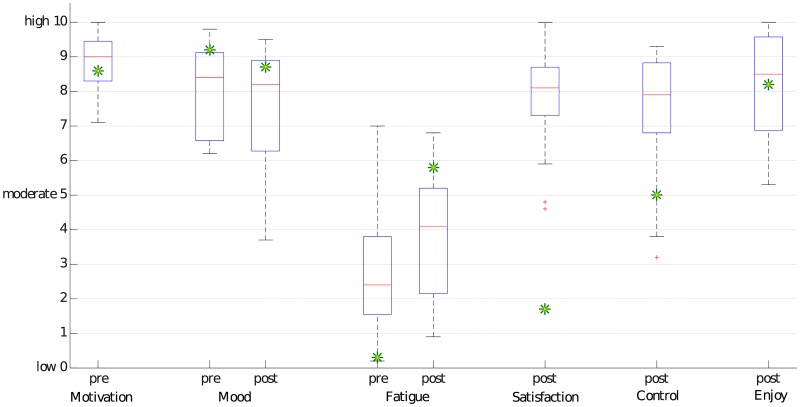
VAS scores. The non-professional participants’ VAS scores are presented as box plots. The professional composer’s scores are shown as green asterisks.

All non-professional participants were highly motivated (M:8.85, SD:0.83). During the study, mood did not change significantly (t(16) = 1.08, p = 0.30, Cohen’s d = 0.26) from M:8.04 (SD:1.31) to M:7.55 (SD:1.66) and fatigue increased significantly from M:2.74 (SD:1.8) to M:3.73 (SD:1.89) (t(16) = 2.52, p = 0.02, Cohen’s d = 0.61). Satisfaction was rated high (M:7.85, SD:1.60). All non-professional participants enjoyed the usage of the brain composing system (M:8.11, SD:1.49) and felt to have good control (M:7.39, SD:1.89). Box plots of the results can be seen in Fig 6.

Ratings of the professional composer are shown as green asterisks in Fig 6. In the satisfaction box plot, the value of the professional composer is an outlier. He argued that the method to make selections restricted his composing process.

#### NASA-TLX

**Fig 7:**
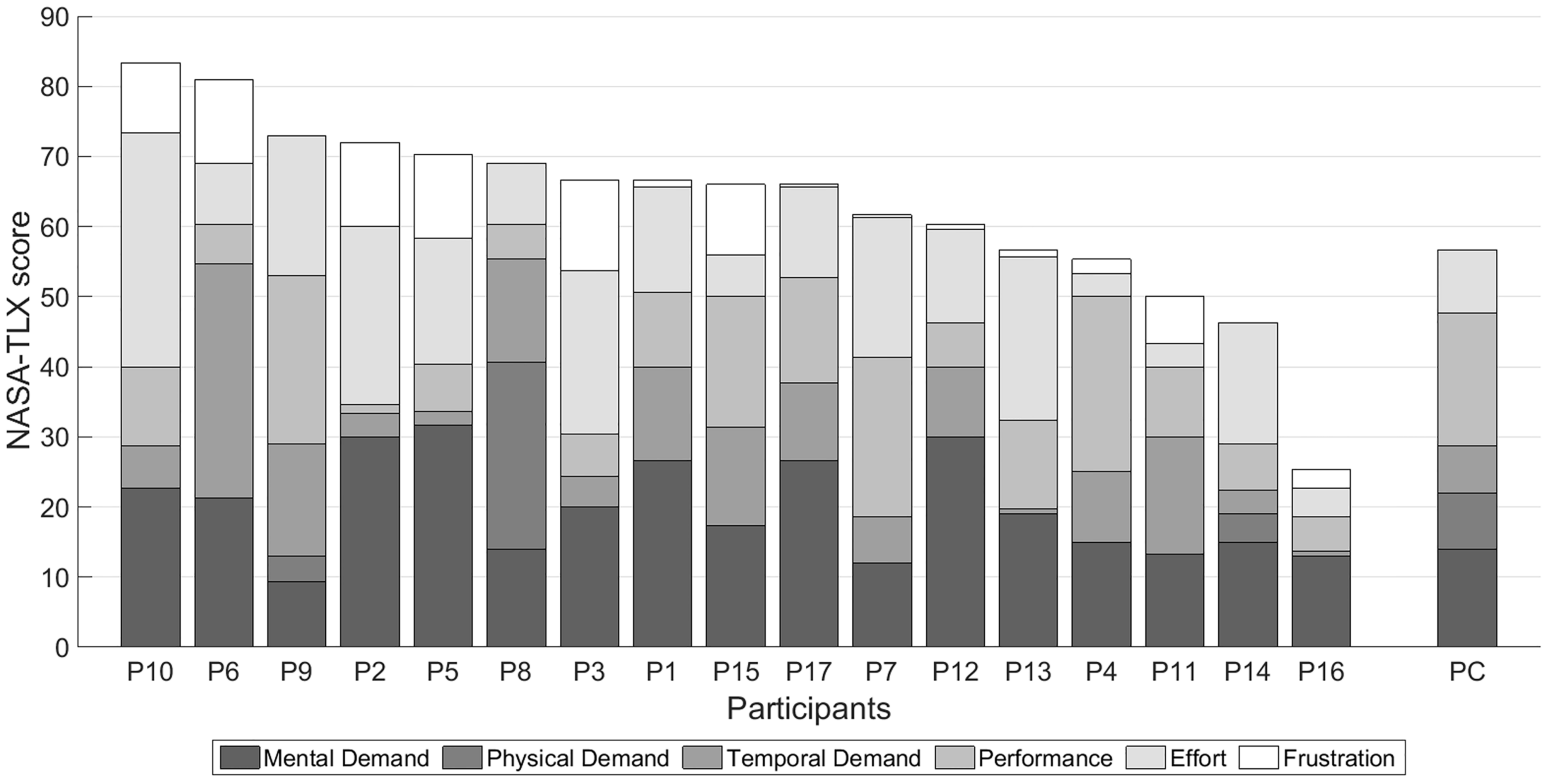
NASA-TLX scores. The non-professional participants’ (P1-17) and the professional composer’s (PC) NASA-TLX scores.

Fig 7 shows the stacked bar plot of the NASA-TLX workload score for all participants. The non-professional participants’ mean global workload score was 62.92 (SD:13.75, range:25.33–83.33). Four participants reached workloads higher than 70. Factors contributing to the global workload score were mental demand (M:19.82, SD:7.07), effort (M:15.06, SD:8.69), performance (M:11.33, SD:7.32), temporal demand (M:9.76, SD:8.14), frustration (M:4.92, SD:5.29), and physical demand (M:2.02, SD:6.48).

The global NASA-TLX workload score of the professional composer was 56.67 (performance: 19.00, mental demand: 14.00, effort: 9.00, physical demand: 8.00, temporal demand: 6.67, and frustration: 0.00).

#### User experience questionnaire

**Fig 8:**
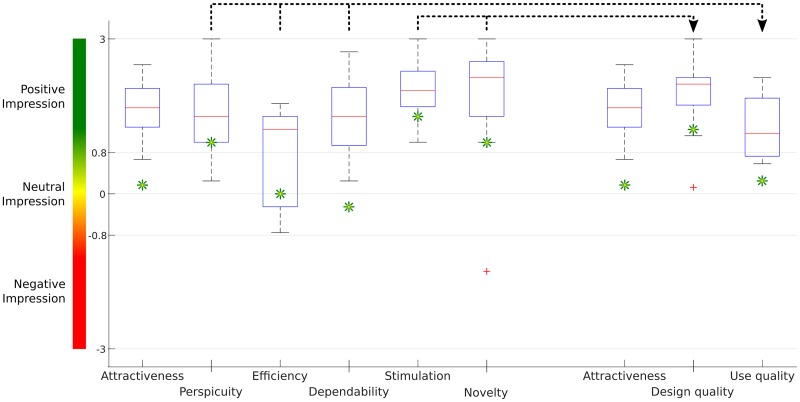
UEQ scores. The non-professional participants’ UEQ scores are presented as box plots. The professional composer’s UEQ scores are shown as green asterisks.

According to the six subscales, the non-professional participants gave the system high average ratings for stimulation (M:2.02, SD:0.58) and novelty (M:1.93, SD:1.09) and a moderate rating for attractiveness (M:1.62, SD:0.50), perspicuity (M:1.60, SD:0.81), efficiency (M:0.84, SD:0.90), and dependability (M:1.49, SD:0.74), see Fig 8. Consequently, the averaged value for the design quality was higher (M:1.97, SD:0.67) than for the user quality (M:1.31, SD:0.60). However, the impression of all parameters was positive except for the efficiency, which was neutral.

The professional composer rated the system lower compared to the other participants’ values, see green asterisks in Fig 8. Participant four rated the “novelty” with a low value (−1.5), see outlier in Fig 8, without giving reasons.

#### Usability questionnaire

Two participants stated that sometimes it was unclear to them where the next note will be set. Normally, the position was indicated by a grey line in the MuseScore software. However, sometimes the note was set before or after this grey line depending on the previous selections. Eight users remarked that they want to have something like a pause button to have time to think about the next step (note) or that the system should detect when they think about the next note and pause automatically. Eight users negatively remarked that the correction of an error can be difficult and often requires more than one selection. The professional composer negatively remarked that it is complicate to select one note and this disturbs his creative process of composing. He suggested that commonly used notes (the combination of note length and pitch) should be selectable with a single selection step to fasten the system.

## Discussion

We presented the implementation and evaluation of the first BCI controlled music composing system. Furthermore, the results indicate that the system works efficiently and effectively and the users enjoyed using it. However, there is still potential to improve the whole system according to the participants’ recommendations. Additionally, a new version of the MuseScore software is available, which solves some arising problems and can be used without substantial changes in the system.

### Composer control method

The communication between the P300-based BCI system and the composing software works only in one direction: from the BCI to the composer. Therefore, if a command from the P300-based BCI does not reach the composing software, an asynchrony between the two systems can occure. For example, if the selection of a different note length is lost between the speller and the composing software, a wrong note length will be displayed in the P300 matrix. This problem can only be solved by a two-way communication between the P300-based BCI and the music composing software. Then the composing software can acknowledge the received commands. The implementation would require a network connection between the applications. Due to the open-source-feature of MuseScore, this implementation would be possible, but with much more implementation effort.

### Evaluation of the BCI efficiency and effectiveness

The used tap water-based EEG amplifier system worked satisfactorily and had the advantage that hair wash was not necessary after the measurements. Excluding the two participants who did not know how to spell at the beginning, the accuracy of the copy-spelling task was above 90%. This high value could not be reached again at the copy-composing or the free-composing task. The copy-composing tasks were more complex and thus cognitively more demanding than the simple spelling tasks. As opposed to copy-spelling, during composing sometimes a combination of subsequent selections was necessary to insert a specific note, i.e., specifying according features such as accidentals or dots. Moreover, in free-composing one needs to focus their attention on the to-be-selected element in the matrix while still creating a composition/melody. For the non-professional participants, this is even more challenging and demanding than copy-composing a given melody. On the other side, it seems that this fact did not influence the performance of the professional composer: his accuracies were at all three P300-based BCI tasks above 93%, see Fig 4. Therefore, one can assume that he had the melody in his mind and just concentrated on the transposition of it. Interestingly, he had lower accuracies when he had to copy-compose a melody than when he composed his own melody.

The pause between the blocks of P300 stimulation sequences was 10 seconds. The professional composer and one non-professional participant told us that 10 seconds were too long. According to their recommendation, the breaks between the P300 stimulation periods could be adapted to the users to increase the efficiency of the system.

Another reason for decreasing accuracies might be the time the participants had to spell in a row. During the spelling task, the participants had a break after five selections. No regular breaks were planned during the composing tasks. Eight of the seventeen non-professional participants recommended that a “pause” element should be included into the P300 matrix to pause the system, cf. [11]. This functionality should definitely be integrated in the next version of the Brain Composing system.

A third reason for the lower composing accuracies could be that nine of the seventeen non-professional participants did not compose music at all and only six of the remaining participants reported to use composing software. Out of this six only one participant solely uses composing software. All the other non-professional participants stated that they first use their favorite instrument to compose and afterwards they transfer the composition to a computer using composing software. Therefore, they are not used to compose directly on the computer like the professional composer.

### Evaluation of behavioral data

The motivation of the users is a crucial factor for P300-based BCIs [51]. The average result of the motivation VAS indicates that all participants were highly motivated. This fact is reflected in the averaged high accuracies. In line with these high accuracies, the participants felt to have good control over the system which, in turn, likely contributed to the high enjoyment and satisfaction they reported. After approximately one hour and 31 minutes of using the system, the fatigue score had increased only slightly from 2.6 to 3.84. This result indicates that the duration of our measurement is not the upper limit of usage and can be extended. One important outlier of the satisfaction values was the score of the professional composer. The way he had to compose music with the Brain Composing system was very different to his normally used method, namely, a musical keyboard in combination with a music composing software (not MuseScore). This combination allows him to give complex commands with low effort. Compared to the Brain Composing method, his method is of course faster and more efficient.

According to the UEQ, the participants had a positive impression of all the asked items, except for efficiency, which was rated as neutral. This is not very surprising, because compared to the normally used healthy participants’ input modalities a BCI works much slower and therefore less efficient. However, one has to keep in mind that the introduced Brain Composing system is not designed for healthy people. It is designed for disabled people, who are not able to use the normal computer input modalities. The design quality factor is very high, which means that the users had a very positive impression about the design of the Brain Composing system. The use quality, which is calculated out of perspicuity, efficiency, and dependability, delivers also a mostly positive impression, albeit with a trend to be neutral. The professional composer rated the attractiveness and dependability significant lower than the other participants, but not negative. The reasons might be the same as for the already described VAS satisfaction item.

Although the given tasks were complex and cognitively demanding, the non-professional participants’ averaged NASA-TLX scores were moderate ranging from 25.33 to 83.33. Mainly three factors contributed to the workload: mental demand, effort, and performance. These three elements have also contributed most to the professional composer’s result. The low values for frustration indicate that the partly low accuracies did not seriously frustrate the participants. The overall rating of the professional composer was lower compared to the mean value of the others. Interestingly, temporal demands did not contribute much to the total score, although eight of the seventeen non-professional participants asked for a “pause” button inside the matrix.

Summarizing the answers from the UQ, many participants recognize that it is very important to avoid errors, because it costs a lot of effort to correct wrong selections. As already mentioned, many users suggest to implement a “pause” button to have flexible time between selections to think or make a break. The most important reported weakness, namely that it was sometimes unclear to the users where the selected note will be set, is already solved and/or integrated in the next version of the MuseScore software as first tests with the new version indicated. There the actual position in the sheet of music is better highlighted with a half transparent grey box instead of a line. Therefore, any uncertainty about the actual composing position should be a problem of the past. Apart from minor remarks, fifteen of the seventeen non-professional participants stated that they enjoyed using the Brain Composing system.

## Conclusion

We could show that it is possible to compose complex music pieces with the introduced Brain Composing system in a fast and comfortable way. The average accuracies of the P300-based BCI tasks were very high even though the participants reported a moderate to high workload. Furthermore, the participants reported that they enjoyed composing with the system.

This was the first step towards establishing a Brain Composing system as a tool for entertainment and, even more important, self-expression for severely disabled people.

## Supporting information

S1 Music Composed music. This mp3 file contains the study participants’ compositions.
(MP3)

S1 Video Brain Composing video. This video shows how the Brain Composing system is used.
(MP4)

S1 File Original raw data of the tasks and the questionnaires. XLSX file containing the original raw data of the tasks and the questionnaires.
(XLSX)
